# An Intriguing Case of Eosinophilia with FIP1L1/PDGFRA Rearrangement Who Presented as Thrombotic Thrombocytopenic Purpura

**DOI:** 10.1155/2019/2820954

**Published:** 2019-10-15

**Authors:** Hassan Alshehri, Mohammad Alnomani, Mubarak Alghamdi, Ibrahim Motabi, Imran Tailor, Nawal Alshehry, Mansour Alfayez, Abdul Rehman Z. Zaidi, Syed Altaf, Azizah AlSwayyed, Ammar AlSughayyer, Syed Z. A. Zaidi

**Affiliations:** ^1^Department of Adult Hematology and BMT, King Fahad Medical City, Riyadh, Saudi Arabia; ^2^Department of Leukemia, The University of Texas MD Anderson Cancer Center, Houston, TX, USA; ^3^Department of Pathology and Clinical Laboratory Medicine, King Fahad Medical City, Riyadh, Saudi Arabia

## Abstract

Myeloid neoplasm with eosinophilia and FIP1-like-1-platelet-derived growth factor receptor-alpha (FIP1L1-PDGFRA) rearrangement is a multi-organ disease with diverse clinical presentation. Thrombotic thrombocytopenic purpura (TTP) is characterized by the concomitant occurrence of often severe thrombocytopenia, microangiopathic hemolytic anemia, and a variable degree of ischemic organ damage. To our knowledge, only one case of eosinophilia with FIP1L1-PDGFRA rearrangement presented as a case of thrombotic thrombocytopenic purpura reported in the literature. We herein report a case of a young male patient with hypereosinophilic syndrome and FIP1L1-PDGFRA rearrangement who presented with asthma, transient ischemic attacks (TIA), and confusion. He had an acquired TTP that was successfully treated with plasma exchanges (PLEX), corticosteroids, rituximab, and later with the addition of imatinib mesylate (Gleevec, Novartis). He remains in complete remission on imatinib 100 mg daily for more than 28 months of follow-up.

## 1. Introduction

Eosinophilia is commonly observed in diverse nonclonal and clonal disorders. In the majority of cases, it is reactive, associated with atopic conditions, autoimmune disorders, and infections or malignancies, while in other instances, it is a result of an underlying hematologic disorder [1].

Current evaluation of suspected hypereosinophilic syndrome (HES) includes a molecular/cytogenetic investigation with either reverse transcription polymerase chain reaction (RT-PCR) or fluorescent in situ hybridization (FISH) for the FIP1L1-PDGFRA rearrangement [2, 3]. It is categorized as a specific entity in the 2008 World Health Organization (WHO) classification of myeloid neoplasms (myeloid and lymphoid neoplasms with eosinophilia and abnormalities of PDGFRA, PDGFRB, and FGFR1) [4, 5]. In 2017 revision of WHO classification, myeloid and lymphoid neoplasms associated with *t* (8; 9) (p22; p24.1) and PCM1-JAK2 have been recognized as provisional entities to this group of eosinophilic disorders [6]. Tyrosine kinase inhibitors, such as imatinib, are effective in the treatment of FIP1L1-PDGFRA mutated disease. Given the striking sensitivity to imatinib, a dose of 100 mg daily is typically sufficient [7–10].

Thrombotic thrombocytopenic purpura (TTP) is characterized by the concomitant occurrence of often severe thrombocytopenia, microangiopathic hemolytic anemia, and a variable degree of ischemic organ damage, particularly affecting the brain, heart, and kidneys [11]. In acquired TTP, the underlying mechanism is deficiency of metalloprotease ADAMTS13 (a disintegrin and metalloproteinase with a thrombospondin type 1 motif, member 13) due to the emergence of its inhibitor.

Simultaneous presentation of HES with FIP1L1-PDGFRA rearrangement and TTP is a unique phenomenon. To our knowledge, only one such a case has been reported in the literature [12].

## 2. Case Presentation

**Figure 1:**
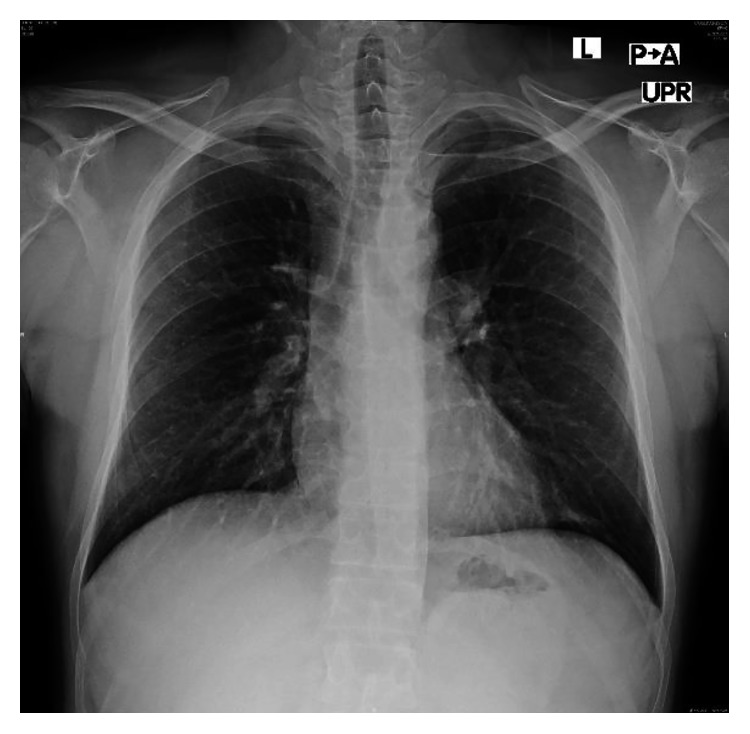
Baseline chest X-ray.

**Figure 2:**
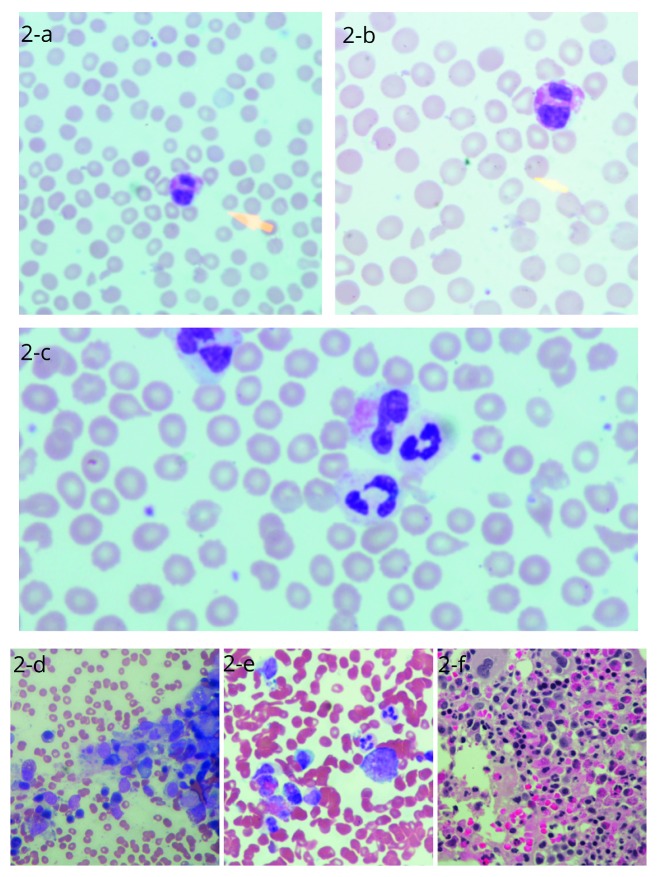
(a, b) Peripheral blood smear revealing schistocytes and eosinophil (May–Grüunwald stain). (c) Partially degranulated eosinophils and schistocytes (May–Grünwald stain). (d, e) Bone marrow aspirate (May–Grünwald stain, 400x magnification). (f) Bone marrow trephine biopsy (hematoxylin-eosin stain, 200x magnification).

A 36-year-old man with past history of 36 pack-years of smoking and bronchial asthma presented to an outside facility on August 11, 2016 with one-hour duration of sudden onset right upper limb weakness that progressed to involve the lower limb along with slurring of speech and altered level of consciousness. CT scan of the brain and carotid ultrasound were reported as normal. His initial complete blood count (CBC) and biochemistry profile showed low hemoglobin and low platelets along with elevated bilirubin. Peripheral blood smear (PBS) showed fragmented red blood cells. TTP was suspected and fresh frozen plasma (FFP) infusion started (due to unavailability of PLEX) along with methylprednisolone intravenously (IV) 500 mg/day. He was then transferred to our facility on August 19, 2016 after 7 days of FFP infusions and methylprednisolone. Upon presentation at our emergency department, he was vitally stable; however, he was intermittently confused with a power of 3/5 in the right upper and lower limbs. His CBC with differential was remarkable for anemia, thrombocytopenia, and leukocytosis with eosinophilia (Table 1). The chest X-ray was normal (Figure 1). Peripheral blood film showed schistocytosis (4%) with eosinophilia in partly degranulated forms (Figures 2(a)–2)(c). ADAMTS13 level and its inhibitor were sent to the reference laboratory; however, due to delay in processing, the sample was denatured and was not analyzable.

As he presented to our facility, PLEX with cryo-poor plasma 1.5 volumes daily was started along with methylprednisolone IV 500 mg/day for 3 days, followed by prednisolone 1 mg/kg/day. The patient's right sided weakness and confusion resolved after 2 sessions of PLEX.

As his eosinophilia persisted after giving pulse steroids, we called for a review of his old records. A blood analysis report of July 2012 had shown normal WBC (5.4 × 10^9^) with normal absolute eosinophil count (0.378 × 10^9^). Workup for eosinophilia including stool examination for ova and parasite, schistosoma serology, ANA, and ANCA was reported negative. Fluorescence in situ hybridization (FISH) screening on peripheral blood for FIP1L1-PDGFRA (4q12) rearrangement was positive in 20.5% of the 200 scored nuclei. T-cell receptor gene rearrangements study was negative.

**Figure 3:**
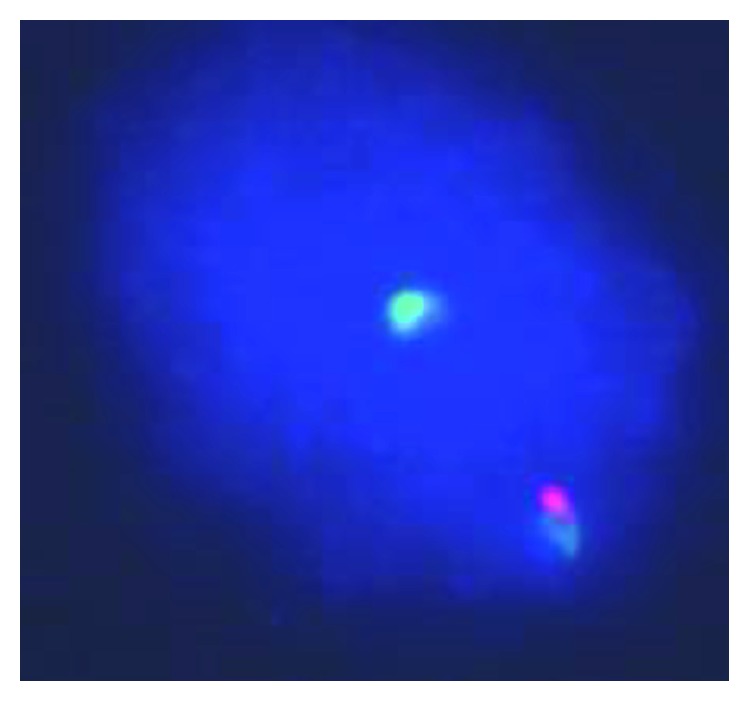
Fluorescence in situ hybridization (FISH) analysis for FIP1L1/CHIC/PDGFRA (using Vysis/Abbott FISH probes) revealed FIP1L1/PDGFRA (4q12) rearrangement in 20.5% of the scored nuclei. ISCN: nuc ish (FIP1L1x2, CHICx1, PDGFRAx2) (FIP1L1 con PDGFRA) (41/200).

Bone marrow aspirate and trephine core biopsies were performed. Bone marrow aspirate showed good spicules, and differential count revealed 2% blasts and 30% Eosinophils. Megakaryocytes were adequate and morphologically unremarkable. Erythroid and myeloid precursors showed progressive and orderly maturation (Figures 2(d) and 2(e)). Trephine core biopsy sections showed cellularity of 90% with trilineage hematopoiesis. Megakaryocytes and erythroid precursors were adequate and morphologically unremarkable. Myeloid precursors were adequate with prominently increased eosinophilic lineage cells (Figure 2(f)). Flow cytometry of the bone marrow (after steroid use) showed a T-cell population (about 78% of the lymphocytes analyzed) with no aberrant loss or aberrant expression of T-cell markers. Normal male karyotype (46, XY) without chromosomal aberrations was noted on the bone marrow specimen. FISH for *t* (9; 22) (q34; q11) and RT-qPCR for BCR : ABL1 p210 was negative. FISH study done on the bone marrow aspirate for FIP1L1/PDGFRA (4q12) rearrangement detected abnormal signals in 26% of the 200 scored nuclei (Figure 3).

After 18 sessions of PLEX, his LDH and platelet normalized. At this point, a trial to weaning off PLEX resulted in re-emergence of schistocytes with elevation in LDH accompanied by a drop-in platelet count, which did necessitate resuming PLEX. Rituximab 375 mg/m^2^ weekly was started and was given for four doses (August 28, 2016, to September 18, 2016). Imatinib 100 mg daily was started on September 26, 2016 for steroid-refractory, FIPL1-PDGFRA rearrangement positive eosinophilia. Rapid resolution of his eosinophilia was noted, and absolute eosinophil count dropped from 3.57 × 10^9^ to 0.14 × 10^9^ after 3 days. In total, 30 PLEX sessions were needed to treat the TTP; the last session was 4 days after the initiation of Imatinib (September 30, 2016).

**Figure 4:**
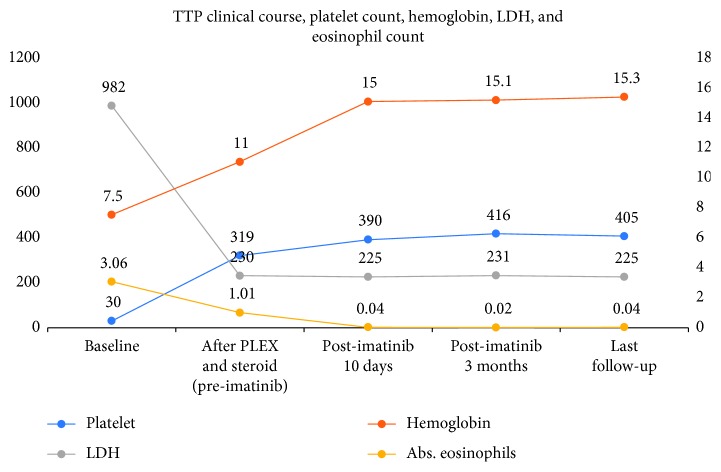
Clinical course, platelet count, hemoglobin, LDH, and eosinophil count of the patient. LDH, lactate dehydrogenase; PLEX, plasma exchange.

After more than 28 months of follow-up, the patient continues to be in remission, with normal eosinophil count and no TTP recurrence (Figure 4).

## 3. Discussion

Acquired TTP is relatively uncommon but a life-threatening disorder. ADAMTS13 deficiency is most frequently acquired via ADAMTS13 autoantibodies, but rarely, it is inherited via mutations of the ADAMTS13 gene. The first acute episode of TTP usually occurs during adulthood, with a predominant anti-ADAMTS13 autoimmune etiology. Rapid recognition of TTP is crucial to initiate appropriate treatment based on daily therapeutic plasma exchange supplying deficient ADAMTS13 (along with the removal of antibody), with or without steroids. Additional immune modulators targeting ADAMTS13 autoantibodies are mainly based on steroids and the anti-CD20 monoclonal antibody rituximab. In refractory or unresponsive TTP, more intensive therapies including twice-daily plasma exchange; pulses of cyclophosphamide, vincristine, or cyclosporine A; or salvage splenectomy may be considered [13]. Multiple reports from Oklahoma [14, 15] and other centers, including our facility, were published [13, 16–19]. There are many acquired associations of TTP; however, the co-occurrence of TTP with HES has rarely been reported, and it is even more unique when TTP-related symptoms are the primary presentation [12]. Chaudhary et al. reported unique association of myeloid neoplasm with eosinophilia and abnormalities of PDGFRA with TTP [12]. Their patient was a female in whom TTP presented as one of the earlier manifestations of myeloproliferative HES with rearrangement of PDGFRA. Their patient was found to have a normal ADAMTS13 level which is not commonly seen with TTP [12]. Earlier than this report, two cases with FIP1L1-PDGFRA-negative hypereosinophilic syndrome and subsequent TTP have been reported [20, 21]. Ohguchi et al. reported a case of HES who subsequently developed TTP due to development of ADAMTS13 inhibitor and was successfully treated with corticosteroids and TPE [20]. Al Aly et al. reported a case who developed idiopathic HES [21]. She was treated with imatinib mesylate and subsequently developed TTP with renal failure. A kidney biopsy was performed and was diagnostic of thrombotic microangiopathy. The patient was treated with TPE and hemodialysis. Her eosinophilia resolved, but she remained dialysis-dependent. Our patient was unique as he had refractory TTP and HES. After 18 days of daily PLEX, an attempt of stopping of PLEX was made. Recurrence with increasing LDH and falling platelet count after 6 days was noted. PLEX was resumed on day 7 along with the addition of rituximab for refractory TTP. Meanwhile, we found evidence of FIP1L1-PDGFRA gene rearrangement by FISH analysis. Hence, we added imatinib on September 26, 2016 at a low dose of 100 mg/day. Absolute eosinophil count normalized within 3 days. This rapid response in eosinophil count can be related to concomitant use of steroids. The patient tolerated imatinib well without any adverse events. PLEX was successfully discontinued after a total of 30 PLEX. The patient was maintained on imatinib 100 mg daily and remains in remission for both TTP and HES after more than 28 months of follow-up.

Hypereosinophilic syndromes (HES) constitute a rare and heterogeneous group of disorders, defined as persistent and marked blood eosinophilia (>1.5 × 10^9^/L for more than six consecutive months) associated with evidence of eosinophil-induced organ damage, where other causes of hypereosinophilia such as allergic, parasitic, and malignant disorders have been excluded [22]. Under normal physiologic conditions, eosinophil production is tightly controlled by the cytokine network. The normal eosinophil count in peripheral blood ranges between 0.05 and 0.5 × 10^9^/L [23]. It can be reactive (secondary) or an integral phenotype of an underlying hematological neoplasm (primary). The diagnosis of clonal eosinophilia requires the demonstration of either a cytogenetic/molecular marker of clonality or bone marrow histological features that are not consistent with an otherwise classified myeloid malignancy [3]. FIP1L1 fusions with PDGFRA, PDGFRB, and FGFR1 were identified in 2002-2003 as recurrent rearrangements, with the FIP1L1-PDGFRA fusion being the most common fusion. Karyotype, although less sensitive, can show corresponding gene rearrangement (4q12 (PDGFRA), 5q31–33 (PDGFRB), or 8p11–12 (FGFR1) [24, 25].

Myeloproliferative neoplasms with FIP1L1-PDGFRA highlight the relevance of tyrosine kinases to both normal cellular physiology and disease states. PDGFRA is a member of type III-receptor tyrosine kinases. FIP1L1/PDGFRA protein fusion leads to deregulated PDGFRA kinase activity and abrogates normal growth factor-dependent stimulation of the receptor [26]. Imatinib at a dose of 100 mg is very effective in this disorder.

The plausible mechanism of the simultaneous presentation of TTP and HES is not clear to us. We ponder if neoantigen form FIP1L1-PDGFRA rearmament stimulated an immune response that cross-reacts with ADAMTS13 resulting in its subsequent depletion, or if this is related to eosinophilia/eosinophilic granules causing an independent platelets aggregation or devouring of ADAMTS13. However, the authors could not demonstrate the deficiency of ADAMTS13 or the presence of autoantibodies against ADAMTS13 due to logistic issues discussed. The eosinophilia in our case was clonal in nature; however, in lymphocyte-variant hypereosinophilia or with Th2/cytokines-mediated eosinophilia, TTP, in theory, can be a manifestation of this autoimmune dysregulation.

## 4. Conclusion

To our knowledge, this is the second case report on the simultaneous presentation of TTP with eosinophilia and FIP1L1-PDGFRA rearrangement. This presentation raises the possibility of a mechanistic association between the two disorders. Low dose imatinib resulted in an excellent and sustained response.

## Figures and Tables

**Table 1:** Laboratory characteristics at baseline, after recovery from TTP post-PLEX and post-imatinib therapy.

Lab parameter	Baseline	After PLEX (pre-imatinib)	Post-imatinib 3 months	Reference range
WBC	13.62	5.78	10.57	3.90–11.00 × 10^9^/L
Abs. neutrophils	7.54	3.7	4.81	1.35–7.50 × 10^9^/L
Abs. lymphocytes	2.32	1.72	5.01	1.50–4.30 × 10^9^/L
Abs. monocytes	0.65	0.35	0.58	0.25–1.00 × 10^9^/L
Abs. eosinophils	3.06	1.01	0.02	0.03–1.00 × 10^9^/L
Abs. basophils	0.05	0.00	0.15	0.25–1.00 × 10^9^/L
Hemoglobin	7.5	11	15.1	13.50–18.00 g/dl
Platelet	30	319	416	155.00–435.00 × 10^9^/L
Coombs test	Negative	Negative	Negative	
Coagulation profile				
aPTT	34.6	31.2	35.2	28.0–41.0 sec
PT	16.5	14.6	13.6	11.5–15.0 sec
INR	1.3	1.1	1.0	0.9–1.2
Fibrinogen	2.6	2.2	4.8	1.4–4.4 g/L
D-dimer	2.4	0.5	0.3	0.0–0.5 *μ*g/mL
Creatinine	110	83	81	62–106 *μ*mol/L
Urea	11.6	7.2	4.4	2.5–6.4 mmol/L
Total bilirubin	94.9	5.2	10.9	0.0–21.0 *μ*mol/L
Direct bilirubin	28.1	1.9	3.2	0.0–3.0 *μ*mol/L
LDH	982	230	231	135–225 U/l
ALT	25	30	35	0–41 U/l
Alkaline phosphatase	90	86	101	40–129 U/l
Albumin	41	34	39	35–50 g/L
Haptoglobin	<0.07			0.3–1.8 g/L
FISH analysis for FIP1L1/PDGFRA (4q12) rearrangement (% of scored nuclei)	26% in bone marrow and 20.5% in peripheral blood	Not done	Negative	Negative

ADAMTS13 level and inhibitor sample were rejected by the reference lab.

## Abbreviations

Abs: Absolute
ADAMTS13: A disintegrin and metalloproteinase with a thrombospondin type 1 motif, member 13
FISH: Fluorescence in situ hybridization
FIP1L1/PDGFRA: FIP1-like-1-platelet-derived growth factor receptor-alpha
LDH: Lactate dehydrogenase
PLEX: Plasma exchange
RDW: Red cell distribution width
MCV: Mean corpuscular volume
MCH: Mean corpuscular hemoglobin.
